# The Levels of Bioelements in Postmenopausal Women with Metabolic Syndrome

**DOI:** 10.3390/nu14194102

**Published:** 2022-10-02

**Authors:** Anna Maria Cybulska, Daria Schneider-Matyka, Mateusz Bosiacki, Dariusz Chlubek, Mariusz Panczyk, Elżbieta Grochans

**Affiliations:** 1 Department of Nursing, Pomeranian Medical University in Szczecin, Żołnierska 48, 71-210 Szczecin, Poland; 2 Department of Functional Diagnostics and Physical Medicine, Pomeranian Medical University in Szczecin, Żołnierska 54, 71-210 Szczecin, Poland; 3 Department of Biochemistry and Medical Chemistry, Pomeranian Medical University in Szczecin, Powstańców Wielkopolskich. 72 St., 70-111 Szczecin, Poland; 4 Department of Education and Research in Health Sciences, Faculty of Health Sciences, Medical University of Warsaw, Litewska 14/16, 00-581 Warsaw, Poland

**Keywords:** bioelements, postmenopausal women, menopause, metabolic syndrome

## Abstract

(1) Metabolic syndrome is a set of factors that considerably increase the risk of developing atherosclerosis, type 2 diabetes, and their cardiovascular complications. Studies show that menopause and the levels of elements may be significantly associated with increased risk of MetS. The present study evaluated the relationship between element levels (Ca, P, Na, K, Fe, Mg, Cu, Zn, Sr) and the incidence of MetS and concomitant metabolic disorders in peri-menopausal women. (2) The study involved 170 perimenopausal women. The methods used were: survey, anthropometric measurement (WC, height, BMI, WHtR), blood pressure measurement, and biochemical analysis of venous blood (lipid profile, glucose, insulin, HbA1C). (3) The study demonstrated statistically significantly higher WC, WHtR, SBP, and DBP values in women with pre-Mets than in those with Mets and the control group. Significantly higher FPG, TG, LDL, HbA1C, insulin, TG/HDL ratio, and TC/HDL ratio were recorded in the MetS group compared to the rest of respondents. In addition, post hoc analysis revealed statistically significant differences in mean K concentrations between pre-MetS and MetS women. (4) Low blood K levels in perimenopausal women are associated with an increased risk of MetS. Significantly higher Cu levels were observed in overweight women. The concentration of Cu negatively correlates with the values of TC, LDL, and SBP.

## 1. Introduction

Metabolic syndrome (MetS) is a cluster of defined metabolic and physical abnormalities, such as visceral obesity, high triglyceride (TG) levels, decreased high-density lipoprotein (HDL) levels, impaired carbohydrate metabolism, carbohydrate intolerance or type 2 diabetes, and hypertension [1]. Both obesity and MetS are characterized by excessive fat deposits in the body. MetS, however, is more than that. It involves the imbalanced intake and expenditure of energy, promoted by low-grade systemic inflammation that results in the aforementioned obesity, hypertension, dyslipidemia, and insulin resistance. In the etiopathogenesis of MetS, an important role is attributed to genetic predisposition, the aging process, endocrine disorders, depression, anxiety, sleep deprivation, chronic stress, and environmental factors [1].

Alarmingly, the American Heart Association considers MetS a global epidemic [2,3] that affects about a quarter of people worldwide and is a significant cause of morbidity and mortality [4]. MetS is widespread in the US [5,6,7] and in Europe [8,9,10], and its incidence increases with age [11,12,13]. As reported in many publications, women are more likely to develop MetS than men [2,9], and menopause is an important risk factor for this condition [14,15]. The aging process differs between the sexes, and changes in the hormonal profile that occur with age are associated with both impaired carbohydrate tolerance and increased cardiometabolic risk. After the onset of menopause, women experience an accumulation of MetS risk factors [16]. A dramatic decrease in estradiol levels at that time is probably the main cause of cardiometabolic diseases in menopausal women [17]. Moreover, menopause entails vascular dysfunction, elevated blood pressure, redistribution of adipose tissue in the abdominal region, and hyperlipidemia [18,19].

Prevention and treatment of MetS in menopausal women require effective management strategies with a focus on lifestyle modifications (i.e., diet and physical activity) [20]. Current evidence suggests that the Mediterranean and vegetarian diets are associated with numerous health benefits, including positive effects on both anthropometric parameters and cardiometabolic risk factors. Moreover, a diet particularly abundant in isoflavones should be integrated into healthy plant-based diets, thus providing nutritional support in the prevention of CVD [21]. Additionally, exogenous natural supplements together with regular physical activity are factors able to protect the body from oxidative damage [21]. Physical activity improves antioxidant defense and lowers lipid peroxidation levels [22]. A review by Król et al. [23] pointed out that a better understanding of the anti-inflammatory properties of stretching may result in increasing its importance in the treatment and recovery of arthritic diseases of various etiologies. Moreover, elderly physically active people show antioxidant activity and lipid peroxidation levels similar to those observed in young sedentary respondents, which confirms the importance of regular physical activity [24]. Regular physical activity is the prime modality for the prevention of numerous diseases e.g., heart failure, and diabetes mellitus [25].

In recent years, there has been an increasing interest in essential trace elements. Many studies indicate a significant association between elemental supplementation and the risk of MetS. There are few studies on the effect of serum element levels on the occurrence of MetS. It is worth noting that elements play an important role in maintaining human metabolic homeostasis [26]. As is well known, any disturbance in the levels of elements significantly disrupts energy metabolism, which may predispose one to obesity-related comorbidities and metabolic changes [27]. Chromium, copper, zinc, and selenium are essential for cardiovascular protection and cholesterol modulation [28,29]. Zinc is a vital component of various enzyme systems (DNA polymerase, glutamate, and lactate dehydrogenase) [30]. According to The Third National Health and Nutrition Examination Survey (NHANES III), magnesium (Mg) deficiency is more common in people belonging to the ‘obese’ BMI category than in the American population with normal weight [31,32]. Many studies show a significant correlation between Mg intake and the risk of MetS [33,34,35,36,37,38,39,40]. Moreover, overweight and obesity are substantially associated with Fe deficiency [34]. In the study by Cybulska et al. [41] there were no significant relationships between serum element levels in menopausal women and their BMI categories. In contrast, according to Han et al. [42], high dietary Ca intake reduces the risk of MetS. The research by Moore et al. [43], on the other hand, suggests that a higher intracellular Ca/Mg ratio, induced by a diet rich in Ca and low in Mg, may result in hypertension, insulin resistance, and MetS. Therefore, it is important to consume the recommended daily intake of Mg and Ca as this may reduce the risk of MetS [44,45].

Some medicines used to treat MetS and comorbidities during menopause may influence the levels of elements. Jurczak et al. [46] observed higher levels of Mg and Zn in the blood plasma of women using menopausal hormone therapy (MHT). Moreover, those women had lower Pb levels in whole blood than the rest of the study participants. Horecka et al. [47] noticed a statistically significant decrease in plasma calcium (Ca) levels in postmenopausal women treated with simvastatin. Certain medications have been shown to contribute to high blood Ca levels (e.g., lithium, diuretics, Ca carbonate, and vitamin D supplements) [48], while long-term medication with drugs such as PPIs or diuretics contributes to Mg deficiency [49].

MetS is a serious health problem in the modern world. Therefore, every effort should be made to build awareness and promote early diagnosis and prevention.

Since the effect of serum element levels on the incidence of MetS in perimenopausal women has not so far been explicitly explained, we decided to assess the relationships between the levels of certain elements (Ca, P, Na, K, Fe, Mg, Cu, Zn, Sr) and the incidence of MetS and concomitant metabolic disorders (diabetes, obesity, hypertension) in this group. We also analyzed the relationships between element levels and the concentrations of lipid parameters (TC, HDL, LDL, TG), carbohydrate metabolism parameters (glucose, insulin levels), as well as anthropometric parameters (BMI, WC, WhtR) and blood pressure (BP).

## 2. Materials and Methods

### 2.1. Organization and Course of Study

Here we present a community-based cohort study of adult Polish women from the West Pomeranian region. It is a cross-sectional, non-randomized study with convenient sampling. Women aged 44–65 years with no history of MHT, no previous psychiatric treatment, no addictions (alcohol), following a normal diet based on Polish cuisine (traditional Polish dishes, such as meat prepared in a variety of ways, bread, potatoes, soup, dumplings, or pickled cabbage) without supplementation with micro- or macronutrients were included in the study. The exclusion criteria were: the use of MHT, psychiatric treatment, alcohol abuse, vegetarian and vegan diets, thyroid disease, cancer, refusal to participate in the study, and improperly completed documentation.

The study was conducted in accordance with the Declaration of Helsinki after obtaining approval from the Bioethics Committee of the Pomeranian Medical University in Szczecin (covered for blind review; permission number: KB-0012/181/13). Recruitment was based on information posters in public places and advertisements in local newspapers. Each respondent gave informed written consent to participate in the study.

This study is part of a larger project intended to assess the health of perimenopausal women living in the West Pomeranian Voivodeship.

### 2.2. Study Design

The study was carried out in four stages using the following methods: survey, anthropometric measurement, blood pressure measurement, and laboratory tests. We used the author’s questionnaire concerning basic sociodemographic data (age, place of residence, employment status, education, marital status), the use of stimulants (alcohol, tobacco), and health (menstruation, inflammation, mental and neoplastic diseases, menopausal status).

### 2.3. Anthropometric Measurements

In the second stage, anthropometric measurements were taken:

- Waist circumference (WC) was measured to the nearest 0.01 m with a flexible measuring tape (SECA) at the level of the navel, as a horizontal distance around the abdomen [50].
- WHtR (waist to height ratio) was calculated using the formula [50]: WHtR = waist circumference (cm)/height (cm).
- Body weight and height were assessed using a certified medical scale with an integrated SECA 711 growth meter, according to a standardized procedure with an accuracy of 0.1 kg and 0.1 cm, respectively. The participants stood with their backs straight, heels together, barefoot, and lightly dressed.
- BMI (body mass index) was calculated using the formula: BMI = weight (kg)/height (m)^2^. BMI (kg/m^2^) was divided into the following categories according to the Center for Disease Control and Prevention (CDC): underweight (BMI < 18.5), normal weight (BMI = 18.5–24.9), overweight (BMI = 25.0–29.9), and obesity (BMI ≥ 30) [51].

### 2.4. RR Measurement

Measurements were taken in accordance with the recommendations for measuring blood pressure [52] and the recommendations of the American Heart Association [53].

### 2.5. Laboratory Analysis

Blood was collected on an empty stomach between 7:00 a.m. and 9:30 a.m. after a 10 min rest, in a sitting position, using a Vacutainer system (BD Vacutainer Eclipse blood collection, BD, New Jersey, USA) by qualified nurses. Blood collection was performed in accordance with the applicable rules and procedures for the collection, storage, and transport of biological material. Biochemical analysis was performed using standard commercial methods in a certified laboratory of the Pomeranian Medical University in Szczecin.

Biochemical parameters, such as insulin, glucose, glycated hemoglobin (HbA1C), total cholesterol (TC), high-density lipoprotein (HDL), and low-density lipoprotein (LDL), as well as triglycerides (TG), were measured.

#### Determination of Serum Ca, P, Na, K, Fe, Mg, Cu, Zn, Sr Levels

The samples were analyzed using inductively coupled plasma optical emission spectrometry (ICP-OES, ICAP 7400 Duo, Thermo Scientific, Waltham, MA, USA) equipped with a concentric nebulizer and a cyclone spray chamber to determine the levels of Ca, P, Na, K, Fe, Mg, Cu, Zn, and Sr. The analysis was performed in radial and axial mode. The samples were thawed at room temperature, and then digested using a CEM MARS 5 microwave digestion system. The volume of the sample given for testing was 0.75 mL. The samples were transferred to clean polypropylene tubes. Then 4 mL of 65% HNO_3_ (Suprapur, Merck, Kenilworth, NJ, USA) was added to each vial, and a 30 min pre-reaction time was allowed for each sample in a clean hood. After the pre-reaction time was completed, 1 mL of unstabilized 30% H_2_O_2_ solution (Suprapur, Merck) was added to each vial. After all reagents were added, the samples were placed in special Teflon vessels and heated in a microwave digestion system for 35 min at 180 °C (15 min. ramp to 180 °C and held at 180 °C for 20 min). After digestion was complete, all samples were taken from the microwave and left to cool to room temperature. In the clean hood, the samples were transferred to acid-washed 15 mL polypropylene tubes. Another 5-fold dilution was made before ICP-OES measurement. A volume of 2 mL was taken from each digestion. The samples were spiked with an internal standard to provide a final concentration of 0.5 mg/L Ytrium and 1 mL of 1% Triton (Triton X-100, Sigma) and diluted to a final volume of 10 mL with 0.075% nitric acid (Suprapur, Merck). The samples were kept in a monitored refrigerator at a nominal temperature of 8 °C until analysis. To prepare blank samples, concentrated nitric acid was added to sample-free tubes and diluted as described above. Multi-element calibration standards (ICP multi-element standard solution IV, Merck, Darmstadt, Germany, phosphorus standard for ICP, Inorganic Ventures, Christiansburg, VA, USA) were prepared with different concentrations of inorganic elements in the same way as the blanks and samples. Deionized water (Direct Q UV, Millipore, Burlington, MA, USA, approximately 18.0 MΩ) was used to prepare all solutions.

Reference material samples (Seronorm ™ Trace Elements Serum L-1, Cat. No. 201405) (n = 3) were prepared in the same way as the samples. The wavelengths (nm) were Ca 315.887; P 178.284; At 589,592; K 766,490; Fe 259.94; Mg 280,270; Cu 224,700; Zn 206,200; and Sr 421.552. The results of the reference material analysis are shown in Table S1.

### 2.6. Distribution of Respondents

In total, 200 perimenopausal women aged 45–65, representing the entire population of the West Pomeranian Voivodeship in the northwestern part of Poland, were recruited for the study. Ultimately 170 female respondents who met all the inclusion criteria (completion rate: 85%) were included in the study.

The size of the study sample was determined based on statistics of the size of the population of women aged 45–64 in the West Pomeranian Voivodeship in 2020 [54]. The confidence level was set at 95%, the maximum error at 7%, and the estimated fraction size at 0.5.

#### 2.6.1. Menopausal Status

The respondents were divided into two groups depending on their menstrual status (presence or absence of menstruation) [55]:

- Perimenopausal respondents: women immediately before menopause with symptoms of impending menopause (when endocrinological, biological, and clinical features of impending menopause begin);
- Postmenopausal respondents: the last menstrual period at least 12 months before the survey.

#### 2.6.2. Metabolic Syndrome

According to the latest criteria proposed by the International Diabetes Federation (IDF) and the modified National Cholesterol Education Program-Adult Treatment Panel III (NCEP-ATP III) [1,56], we assumed that three of five risk factors are needed to diagnose MetS in women. These include:

- WC ≥ 80 cm, TG > 150 mg/dL (1.7 mmol/L) or specific treatment of this lipid abnormality;
- HDL < 50 mg/dL (1.3 mmol/L) or specific treatment of this lipid abnormality;
- Elevated blood pressure (BP): systolic BP ≥ 130 or diastolic BP ≥ 85 mmHg or treatment of previously diagnosed hypertension;
- Elevated fasting plasma glucose (FPG) level ≥ 100 mg/dL (5.6 mmol/L) or previously diagnosed type 2 diabetes. If it is above 5.6 mmol/L or 100 mg/dL, an oral glucose tolerance test (OGTT) is strongly recommended, but it is not necessary to determine the presence of the syndrome.

The women were defined as having pre-metabolic syndrome (pre-MetS) if they had at least two components of MetS but did not meet the above criteria for MetS diagnosis [57].

The women were divided into three groups:

- MetS group: women with MetS;
- Pre-MetS group: women at risk of MetS;
- Control group: women without MetS and the risk of MetS.

#### 2.6.3. Hypertension

Based on the 2019 guidelines of the Polish Society of Hypertension, as well as those of the European Society of Cardiology (ESC) and the European Society of Hypertension (ESH), hypertension was diagnosed if the patient had systolic blood pressure (SBP) ≥ 140 mmHg or diastolic blood pressure (DBP) ≥ 90 mmHg [58] or reported having been prescribed antihypertensive drugs.

#### 2.6.4. Obesity

Based on the recommendations of the Centers for Disease Control and Prevention (CDC), the following ranges were established [51]: underweight (BMI < 18.5), normal weight (BMI = 18.5–24.9), overweight (BMI = 25.0–29.9), and obesity (BMI ≥ 30). Additionally [59]:

- Abdominal obesity (central obesity) was defined as waist circumference ≥ 88 cm and ≥ 102 cm for women and men, respectively;
- General obesity was defined as BMI ≥ 30 kg/m^2^.

### 2.7. Statistical Analysis

Quantitative, nominal, and ordinal variables were described using descriptive statistical methods. The measures determined for quantitative variables were: central tendency (mean, M) and dispersion (standard deviation, SD), and for nominal and ordinal variables, number (N) and frequency (%).

Cross-tables and Pearson’s chi-squared test with odds ratio were used to assess the frequency of difference for variants of categorical variables. To assess differences (perimenopause vs. postmenopause) for selected quantitative variables, Student’s t-tests were employed, and mean differences were calculated.

All calculations were performed with Statistica^TM^ 13.3 software (TIBCO Software, Palo Alto, Santa Clara, CA, USA). For all analyses, *p* < 0.05 was considered statistically significant.

## 3. Results

### 3.1. Baseline Clinical Characteristics and Laboratory Results

Table 1 and Table 2 show the anthropometric and clinical characteristics of the respondents with regard to MetS; 26.5% of the women were diagnosed with pre-MetS, and 9.41% were diagnosed with MetS.

We compared the groups (control, pre-MetS, MetS) in terms of the results of anthropometric measurements and clinical characteristics. Statistically significant differences were observed in WC, WHtR, SBP, and DBP values. Pre-Mets subjects had significantly higher WC, WHtR, SBP, and DBP values than those from the Mets and the control groups (Table 1 and Table 2).

Post hoc analysis revealed statistically significant differences in the mean WC values between the pre-MetS group and the control group (*p* = 0.0001). Moreover, there were statistically significant differences in the mean WHtR values between the pre-MetS group and the control group (*p* = 0.00006). Additionally, analysis demonstrated statistically significant differences in the mean SBP values between the pre-MetS group and the control group (Dunnett’s test, *p* = 0.00002) and between the MetS group and the control group (*p* = 0.0009). There were also statistically significant differences in the mean DBP values between the pre-MetS group and the control group (*p* = 0.00002) and between the MetS group and the control group (*p* = 0.002) (Table S2).

We compared the levels of biochemical parameters between the groups (control, pre-MetS, MetS). There were statistically significant differences in the levels of FPG, TG, HDL, HbA1C, insulin, TG/HDL ratio, TC/HDL ratio, and LDL/HDL ratio between the respondents. Significantly higher values of FPG, TG, LDL, HbA1C, insulin, TG/HDL ratio, and TC/HDL ratio were noted in the MetS group compared to the rest of the respondents (pre-Mets and control). On the other hand, higher HDL values were noted in the subjects from the control group (Table 3).

Post hoc analysis showed statistically significant differences in the mean FPG levels between the pre-MetS group and the control group (*p* = 0.0439) and between the MetS group and the control group (*p* = 0.003).

In addition, there were statistically significant differences in the mean TG levels between the pre-MetS group and the control group (*p* = 0.002), and between the MetS group and the control group (*p* = 0.00002).

There were also statistically significant differences in the mean values of HDL (mg/dL) between the pre-MetS group and the control group (*p* = 0.0247), and between the MetS group and the control group (*p* = 0.00002).

Post hoc analysis showed statistically significant differences in the mean TG/HDL ratios between the pre-MetS group and the control group (*p* = 0.0156), and between the MetS group and the control group (*p* = 0.00002). There were also statistically significant differences in the mean TC/HDL ratios between the pre-MetS group and the control group (*p* = 0.0001), and between the MetS group and the control group (*p* = 0.00002). We also noted statistically significant differences in the mean LDL/HDL ratios between the pre-MetS group and the control group (*p* = 0.0014), and between the MetS group and the control group (*p* = 0.00002) (Table S3).

### 3.2. Correlations between the Levels of Bioelements (Ca, P, Na, K, Fe, Mg, Cu, Zn, Sr) and MetS, Menopausal Status, BMI, and HA

We determined selected element blood levels. Analysis of the data showed that the mean element levels in the blood of the examined women were within the normal range, but in some cases, they were below or above the norm (Table 4 and Table S2).

Analysis of the levels of selected elements (Ca, P, Na, K, Fe, Mg, Cu, Zn, Sr) in relation to the menopausal status (perimenopause, postmenopause) showed no statistically significant relationships (Table 5). 

In the study, we analyzed the relationships between element levels (Ca, P, Na, K, Fe, Mg, Cu, Zn, Sr) and MetS. A statistically significant relationship was observed between K levels and the presence of MetS. Women with pre-MetS had significantly higher K values than other respondents (*p* = 0.024). Post hoc analysis showed statistically significant differences in the mean K levels between women with pre-MetS and MetS (*p* = 0.0097) (Table 6).

We analyzed element concentrations (Ca, P, Na, K, Fe, Mg, Cu, Zn, Sr) in relation to BMI (normal, overweight, obesity). We noted a statistically significant relationship between Na, Mg, and Cu levels and BMI values. Significantly higher Na and Mg concentrations were noted in normal-weight women. Higher Cu levels, on the other hand, were recorded in overweight women (Table 7). Post hoc analysis showed statistically significant differences in the mean Na and Mg levels between normal-weight and overweight women (*p* = 0.008 and *p* = 0.008, respectively). In addition, post hoc analysis showed statistically significant differences in the mean Cu levels between overweight and obese women (*p* = 0.017) (Tables S4 and S5).

The levels of selected elements (Ca, P, Na, K, Fe, Mg, Cu, Zn, Sr) were analyzed in relation to the presence of hypertension. No statistically significant relationships were observed between the levels of the elements and the presence of hypertension (Table 8).

### 3.3. Relationships between the Concentrations of Elements (Ca, P, Na, K, Fe, Mg, Cu, Zn, Sr) and the Concentrations of Lipid Parameters (TC, HDL, LDL, TG), Parameters of Carbohydrate Metabolism (Fasting Glucose, Insulin), as well as Anthropometric Parameters (BMI, WC, WhtR) and Blood Pressure (BP)

In this study, we assessed the relationship between the concentrations of elements (Ca, P, Na, K, Fe, Mg, Cu, Zn, Sr) and the concentrations of lipid parameters (TC, HDL, LDL, TG), parameters of carbohydrate metabolism (fasting glucose, insulin), as well as anthropometric parameters (BMI, WC, WhtR) and blood pressure (BP).

Based on the results, we observed statistically significant correlations between selected elements and selected lipid parameters, carbohydrate metabolism parameters, as well as WC and blood pressure.

The study revealed a positive correlation between the levels of FPG and Sr (r = 0.16, *p* = 0.041), and a positive correlation between the levels of HbA1C and Sr (r = 0.18, *p* = 0.017). There was also a negative correlation between the levels of TC and Cu (r = −0.18, *p* = 0.022). Additionally, we observed statistically significant correlations between the levels of LDL and Na (r = −0.15, *p* = 0.045), and the levels of LDL and Cu (r = –0.16, *p* = 0.042). WC negatively correlated with the level of Mg (r = −0.16, *p* = 0.035). Statistically significant correlations were also noted between SBP and Na (r = –0.19, *p* = 0.011), SBP and Mg (r = −0.23, *p* = 0.003), and SBP and Cu (r = −0.17, *p* = 0.029). No statistically significant correlations were observed between the other variables and the rest of the elements analyzed in this study (Table 9).

## 4. Discussion

Obesity and comorbidities, including MetS and type 2 diabetes, are a significant medical problem worldwide. The incidence of cardiometabolic diseases is rapidly increasing around the world, which is why early identification of risk factors and the introduction of preventive measures are so important. Many researchers are increasingly recognizing the significant impact of diet, and thus serum element levels on the risk of MetS in the population.

Moreover, literature data suggest that the gut microbiota may affect host metabolism and thus influence several MetS risk factors. Dysbiosis is a perturbation of the gut microbiota’s composition and activity and is involved in the etiopathogenesis of multiple chronic diseases [60]. It is important to find the correlation between different dietary strategies and changes in gut microbiota-derived metabolites. In our research, we mainly focused on the relationships between the levels of certain elements (Ca, P, Na, K, Fe, Mg, Cu, Zn, Sr) and the incidence of MetS and concomitant metabolic disorders, such as diabetes, obesity, and hypertension. In the future, we will explore the influence of the gut microbiota and specific dietary patterns on the reduction of inflammatory processes and negative consequences associated with MetS.

### 4.1. Correlation between the Levels of Bioelements (Ca, P, Na, K, Fe, Mg, Cu, Zn, Sr) and Factors such as MetS, Menopausal Status, BMI, and HA

Our research showed a statistically significant relationship between the concentration of K and the presence of MetS. Women with pre-MetS had significantly higher K levels than other respondents (*p* = 0.024). No statistically significant relationships were observed between the remaining elements and the occurrence of MetS. Moreover, no statistically significant relationships were found between element levels and menopausal status.

In a study by Lee et al. [61], higher dietary K intake was shown to be an important determinant of risk for MetS and insulin resistance. This relationship was particularly pronounced among postmenopausal women compared to premenopausal women. A number of studies indicate the link between K intake and the risk of MetS [62]. In a study by Sun et al. [63] low serum potassium level was significantly associated with prevalence of metabolic syndrome in middle-aged and elderly Chinese. Murao et al. [64] showed that in drug-naïve normotensive individuals, the effects of a higher-potassium diet and IR on blood pressure were more evident in women. They suggested that to prevent the new onset of hypertension and its complications, the balances of a sodium restriction and an increased potassium intake are important, even in normotensive individuals, independent of known risk factors for salt sensitivity, especially in women. Aburto et al. [65] reported that high intake of dietary K was inversely related with blood pressure and suggested that potassium intake affects MS prevalence. Teramato et al. [66] showed that low potassium intake was significantly related to increased SBP and DBP. A significantly higher prevalence of MetS was found in women in the lowest quartile of potassium intake compared with the higher quartiles. A study by Cai et al. [67] indicated that serum potassium was associated with obesity, and potassium intake was associated with metabolic syndrome. This research also demonstrated a protective effect of adequate potassium intake on obesity and metabolic syndrome.

Han et al. [42] noted that high dietary Ca intake reduces the risk of MetS. In contrast, a study by Moore et al. [43] suggested that a higher intracellular Ca/Mg ratio, induced by a diet rich in Ca and low in Mg, may result in hypertension, insulin resistance, and MetS. Therefore, it is important to consume the recommended daily intake of Mg and Ca, as this may reduce the risk of MetS [44,45]. In contrast, a study by Lin et al. [68] showed that postmenopausal patients had higher total serum Ca levels than premenopausal patients (2.31 ± 0.14 vs. 2.26 ± 0.12 mmol/L; *p* = 0.009). What is more, total serum Ca levels were higher in women aged >55 than in those aged ≤55 (2.32 ± 0.13 vs. 2.28 ± 0.12 mmol/L; *p* = 0.023). This is an interesting finding, because postmenopausal women usually suffer from estrogen deficiency, which increases the risk of osteoporosis due to Ca resorption from bones [69]. This may be one reason why postmenopausal patients had higher serum Ca levels than the premenopausal group. Pardhe et al. [70], LeKhi et al. [71], and Onyeukwu et al. [72] reported that serum Ca levels in postmenopausal women were significantly lower than in premenopausal women. 

It is worth noting that Luciano-Mateo et al. [27] observed reduced levels of Ca, Fe, Mg, Na, and Zn and increased levels of some trace elements in serum (e.g., Sr) in women with giant obesity. In a study by Lu et al. [73], higher serum levels of Zn, Cu, and Fe were associated with the risk of MetS. In addition, serum levels of Zn, Cu, and Fe increased with a rise in the number of metabolic factors (*p* < 0.001).

The IMMIDIET study in Europe and the NHANES study in Korea [74] found no association between serum Zn levels and MetS. In contrast, Lu et al. [73] reported a strong positive association between Zn and MetS, suggesting impaired Zn homeostasis in patients with MetS in addition to insulin resistance and obesity.

Eshak et al. [75] and Choi et al. [36] found Cu to be a protective factor that was negatively associated with MetS incidence, as did Seo et al. [74], Seet et al. [76], and Fang et al. [77], who reported negative correlations between serum Cu levels and MetS. Different results were obtained by Lu et al. [78], whose study showed that elevated serum Cu concentrations were associated with an increased risk of MetS.

Our research showed a statistically significant relationship between Na, Mg, and Cu levels and BMI values. We noted that Na and Mg levels were significantly higher in normal-weight women. In contrast, overweight women had higher Cu levels. No statistically significant relationships were observed between the other elements and BMI values.

According to the NHANES III, Mg deficiency is more common in obese individuals than in the American population with normal weight [31,32]. Similarly, Mg intake is impaired in 35% of French people with BMI > 35 kg/m^2^ [79]. Moreover, a review of the literature showed positive correlations between low dietary Mg intake and the risk of cardiometabolic diseases regardless of other risk factors, such as age, sex, BMI, race, education, marital status, smoking, alcohol consumption, physical exercises, and the use of antihypertensive or lipid medications [33,34,35,36]. Dibaba et al. have proved that dietary Mg intake is inversely proportional to the incidence of MetS [37]. Similar findings were presented by Song et al. [33] who studied 11,000 middle-aged and elderly women. They observed that high dietary Mg intake reduced systemic inflammation and the risk of MetS. Jang et al. [39], on the other hand, observed an inverse correlation between dietary Mg intake and MetS incidence in their study of the Chinese population. Additionally, the Coronary Artery Risk Development in Young Adults (CARDIA) study indicated that Mg intake is inversely related to the incidence of obesity and C reactive protein levels [73]. Thus, there is a significant relationship between Mg intake and obesity markers, such as BMI and waist circumference [37,80,81,82,83], as well as the risk of cardiovascular disease, diabetes, and overall mortality [82,83,84,85,86]. In a study by Luciano-Mateo et al. [27], Mg levels were negatively correlated with blood glucose levels in obese women. This is consistent with the reports by Oliveira et al. [87], who confirmed reduced serum Mg levels in obese individuals. Additionally, Cybulska et al. [41] showed that BMI may be one of the factors influencing the relationship between serum element levels in women, especially during menopause. Higher levels of circulating Mg are associated with a lower risk of cardiovascular disease, including coronary artery disease [88], while low chronic dietary Mg intake leads to both serum and intracellular Mg deficiency, which is particularly pronounced in obese individuals with MetS and in the elderly [89,90,91]. As said by Sachinidis et al. [92], serum ferritin levels increase in proportion to the degree of insulin resistance and the number of MetS components. In turn, dysmetabolic iron overload syndrome (DIOS) heralds the onset of type 2 diabetes and non-alcoholic liver disease (NAFLD). Elevated serum Fe levels [93] in the diet are associated with an increased risk of diabetes. A study by Lu et al. [68] confirmed that elevated serum Fe levels are strongly correlated with MetS.

A study by Yun et al. [94] showed that the risk of Fe deficiency in postmenopausal women was lower than in premenopausal women (PR = 0.813, 95%CI 0.668, 0.998, *p* = 0.038). The risk of MetS was 2.562 times lower in premenopausal women with Fe deficiency than in those without it (PR = 0.390, 95%CI 0.266, 0.571, *p* < 0.001). Conversely, in the group of postmenopausal women, the risk of MetS was higher among those deficient in Fe than among those without such a deficiency (PR = 1.849, 95%CI 1.406, 2.432, *p* < 0.001). The risk of MetS increased in both premenopausal and postmenopausal women with an increase in serum ferritin levels. A study by Pitchika et al. [95] suggests that there is an association between ferritin and type 2 diabetes and MetS, which can be partially explained by liver dysfunction.

### 4.2. Relationships between the Concentrations of Elements (Ca, P, Na, K, Fe, Mg, Cu, Zn, Sr) and the Concentrations of Lipid Parameters (TC, HDL, LDL, TG), Parameters of Carbohydrate Metabolism (Fasting Glucose, Insulin), as well as Anthropometric Parameters (BMI, WC, WhtR) and Blood Pressure (BP)

In our study, FPG levels were positively correlated with Sr levels (r = 0.16; *p* = 0.041). Furthermore, a positive correlation was observed between HbA1C and Sr levels (r = 0.18; *p* = 0.017). A negative correlation was noted between TC values and Cu levels (r = −0.18; *p* = 0.022). Statistically significant correlations were also observed between the levels of LDL and Na (r = −0.15; *p* = 0.045), and LDL and Cu (r = −0.16; *p* = 0.042). There was a negative correlation between WC and Mg levels (r = −0.16; *p* = 0.035). Statistically significant correlations were also observed between SBP and Na (r = −0.19; *p* = 0.011), SBP and Mg (r = −0.23; *p* = 0.003), and SBP and Cu (r = −0.17; *p* = 0.029). No statistically significant correlations were observed between the other variables and the rest of the elements analyzed in our study (Table 8).

A study by Lin et al. [68], on the other hand, showed that total serum Ca levels were significantly positively correlated with FPG (*p* = 0.035), TG (*p* = 0.011), HDL (*p* = 0.002), LDL (*p* < 0.001), and TC (*p* < 0.001). HDL (*p* = 0.027), LDL (*p* = 0.013), and TC (*p* = 0.003) tended to increase with increasing corrected serum Ca concentrations, while FPG (*p* = 0.638) and TG (*p* = 0.075) did not show an increasing trend.

Studies by Yamaguchi et al. [96] and Sun et al. [97] showed that serum Ca levels were independently positively correlated with FPG in both subjects with and without diabetes. They also found that albumin-adjusted serum Ca levels were related to total cholesterol (TC) and high-density lipoprotein (HDL) in women [98]. 

In a study by Lu et al. [37], Zn was positively associated with elevated fasting glucose, BP, and triglycerides after adjusting for BMI and insulin resistance. Moreover, these authors observed that elevated Cu levels were positively correlated with elevated fasting glucose levels and low HDL-C. Only a cross-sectional study in Lebanon demonstrated a positive association between serum HDL-C and Cu [99]. 

Hua et al. [100] reported a significant positive association of serum Ca2+ levels with the incidence of hypertension. This association was independent of variables such as age, sex, race, education level, BMI, history of diabetes, smoking, alcohol consumption, sodium intake, triglycerides, total cholesterol, albumin, eGFR, and serum phosphorus. In turn, many previous studies have shown a statistical correlation between low Ca levels and a higher risk of elevated blood pressure [101,102,103]. Meta-analyses by Cormick et al. [104] and Dickinson et al. [105] confirmed that Ca supplementation can lower systolic blood pressure. This is consistent with the results obtained by Han et al. [42], who provided evidence that Ca supplementation may have a putative protective effect against MetS. They found that increasing dietary Ca intake by 300 mg/day was significantly associated with a 7% decrease in MetS risk. 

Cho et al. [106] assert that menopause may play a significant role in the protective effect of Ca on the occurrence of MetS. They found that only in postmenopausal women were higher levels of Ca intake associated with a reduced risk of MetS. Drouillet et al. [107] observed that an increase in the quartile of dietary Ca intake was accompanied by a decrease in insulin levels and blood pressure, and an increase in HDL cholesterol in women.

Given a higher prevalence of MetS in postmenopausal women, and their elevated risk of developing cardiovascular disease and type 2 diabetes, women should be monitored for these conditions and informed about the importance of healthy lifestyles to reduce morbidity and early mortality in this group.

## 5. Conclusions

- Low blood K levels in perimenopausal women are associated with increased risk of MetS. It is therefore important to treat K deficiencies through dietary K intake to reduce the risk of MetS among perimenopausal women. Further research is needed to determine whether K supplementation is an effective solution in reducing the risk of MetS among women.
- Significantly higher levels of Cu were observed in overweight women. Moreover, the concentration of Cu negatively correlated with the values of TC, LDL, and SBP. This finding shows that there are links between trace element levels and metabolic risk in perimenopausal women. However, more research is required to elucidate the causal relationship between trace element levels and metabolic risk in women.

## 6. Limitations

This study has some limitations that should be noted:

- The biggest limitation of the study is a relatively small number of patients in both groups. This was mainly due to the strict inclusion and exclusion criteria.
- In this study, we did not specify dietary factors, and we excluded micronutrient supplementation.
- The duration of elevated BP, glucose intolerance, and dyslipidemia has not been reported in patients with and without MetS, suggesting a potential impact of the trajectory of cardiometabolic diseases on the change in serum micronutrient levels over time.
- The lack of menstruation for at least 12 months was diagnosed on the basis of gynecological history but was not confirmed by the measurement of FSH levels. Both of these variables may have influenced the results of our study.
- The study does not include liver function parameters. This is a major limitation, given that NAFLD is an important correlate of MetS.

## Figures and Tables

**Table 1:** Baseline data of menopausal women from the MetS, pre-MetS, and control groups.

Variables	Pre-MetS (n = 45)		MetS (n = 16)		Control (n = 109)		F (2,167)	*p* *	η^2^ (95%CI)
	M	SD	M	SD	M	SD			
Age (years)	54.71	5.42	54.44	5.15	54.12	5.03	0.217	0.805	-
WC (cm)	96.18	10.69	93.50	7.31	86.73	14.60	8.898	<0.001	0.096 (0.024; 0.181)
Body mass (kg)	76.28	12.77	77.09	11.19	72.71	15.99	1.292	0.277	-
Height (cm)	163.29	5.35	164.94	6.06	164.39	6.30	0.682	0.507	-
BMI (kg/m^2^)	28.58	4.47	28.36	3.97	26.82	5.11	2.408	0.093	-
WHtR (%)	0.59	0.067	0.57	0.055	0.53	0.09	9.366	<0.001	0.101 (0.026; 0.186)
SBP (mmHg)	128.04	18.06	124.63	17.93	111.30	12.56	22.970	<0.001	0.216 (0.110; 0.313)
DBP (mmHg)	83.62	9.34	81.94	9.02	74.22	9.02	19.133	<0.001	0.186 (0.086; 0.282)

WC–waist circumference, BMI–body mass index, WHtR–waist/height ratio, SBP–systolic blood pressure, DBP–diastolic blood pressure, * one-way ANOVA.

**Table 2:** Baseline data of menopausal women from the MetS, pre-MetS, and control groups.

Variables	Control (n = 109)		Pre-MetS (n = 45)		MetS (n = 16)		χ^2^_df=2_	*p* *
	n	%	n	%	n	%		
Obesity	26	23.85	14	31.11	4	25.00	0.882	0.643
Abdominal obesity	51	46.79	39	86.67	15	93.75	29.099	<0.001
General obesity	29	26.61	17	37.78	4	25.00	2.080	0.353
Current smoking	23	21.10	4	8.89	5	31.25	4.893	0.087
Hypertension	7	6.42	24	53.33	8	50.00	46.965	<0.001

* Pearson’s chi-squared test.

**Table 3:** Biochemical parameters of the studied women with regard to MetS.

Variables	Pre-MetS (n = 45)		MetS (n = 16)		Control (n = 109)		F (2,167)	*p* *	η2 (95%CI)
	M	SD	M	SD	M	SD			
FPG (mg/dL)	90.68	34.81	99.99	25.88	83.29	8.68	5.613	0.004	0.062 (0.007; 0.138)
TG (mg/dL)	109.16	53.82	168.26	74.36	85.56	29.86	27.501	<0.001	0.248 (0.138; 0.345)
TC (mg/dL)	217.77	33.01	223.38	37.06	210.82	31.18	1.515	0.223	-
HDL (mg/dL)	67.11	20.41	51.04	14.76	73.87	15.55	13.577	<0.001	0.140 (0.051; 0.232)
LDL (mg/dL)	125.98	38.13	138.69	26.00	119.77	27.58	2.921	0.057	-
HbA1C (%)	5.43	0.63	5.82	0.85	5.28	0.27	9.717	<0.001	0.104 (0.028; 0.190)
Insulin (µlu/mL)	11.12	8.67	12.66	6.03	7.89	4.16	7.967	<0.001	0.087 (0.019; 0.169)
TG/HDL ratio	1.85	1.18	3.58	2.18	1.32	1.05	24.130	<0.001	0.224 (0.117; 0.321)
TC/HDL ratio	3.52	1.12	4.54	0.81	2.95	0.64	30.398	<0.001	0.267 (0.155; 0.364)
LDL/HDL ratio	2.11	0.97	2.83	0.58	1.70	0.57	20.323	<0.001	0.196 (0.094; 0.292)

TG–triglyceride, TC–total cholesterol, HDL–high-density lipoprotein, LDL–low-density lipoprotein.

**Table 4:** The levels of bioelements in the whole blood and serum of the participants.

Element	M	SD	Mdn	IQR/2	Min	Max
Ca (mg/L)	103.28	28.71	98.35	21.94	52.49	244.85
P (mg/L)	110.68	38.85	104.82	19.03	51.17	357.73
Na (mg/L)	2271.05	709.09	2225.36	391.82	839.98	6551.39
K (mg/L)	394.63	308.61	263.42	180.26	62.02	1415.86
Fe (mg/L)	1.59	0.63	1.45	0.40	0.38	3.84
Mg (mg/L)	18.51	6.37	17.66	3.57	9.10	54.74
Cu (mg/L)	1.09	0.43	1.05	0.26	0.36	3.25
Zn (mg/L)	2.56	1.45	2.18	0.76	0.63	9.25
Sr (mg/L)	0.14	0.07	0.12	0.04	0.03	0.34

M—mean, SD—standard deviation, Mdn—median, IQR/2—interquartile range.

**Table 5:** The levels of bioelements with regard to menopausal status (perimenopause and menopause).

Bioelement	Perimenopause (n = 42)		Postmenopause (n = 128)		t_df = 168_	*p* *	Δ	95%CI
	M	SD	M	SD				
Ca (mg/L)	101.93	27.30	103.72	29.25	−0.349	0.728	1.78	−8.32; 11.89
P (mg/L)	107.43	37.09	111.75	39.49	−0.625	0.533	4.33	−9.34; 17.99
Na (mg/L)	2302.24	746.61	2260.82	699.08	0.328	0.744	−41.42	−291.02; 208.17
K (mg/L)	371.50	310.18	402.22	308.94	−0.559	0.577	30.72	−77.84; 139.28
Fe (mg/L)	1.50	0.56	1.62	0.66	−1.058	0.291	0.12	−0.10; 0.34
Mg (mg/L)	18.64	7.61	18.47	5.95	0.148	0.883	−0.17	−2.41; 2.08
Cu (mg/L)	1.15	0.42	1.08	0.43	0.915	0.362	−0.07	−0.22; 0.08
Zn (mg/L)	2.47	1.56	2.59	1.41	−0.435	0.664	0.11	−0.40; 0.62
Sr (mg/L)	0.13	0.05	0.15	0.07	−1.003	0.317	0.01	−0.01; 0.04

M—mean, SD—standard deviation.

**Table 6:** The levels of bioelements in women with and without MetS.

Bioelement Levels	MetS (IDF)						F (2, 167)	*p* *	η2 (95%CI)
	Pre-MetS (n = 45)		MetS (n = 16)		Control (n = 109)				
	M	SD	M	SD	M	SD			
Ca (mg/L)	100.37	25.78	99.74	28.45	104.99	29.97	0.543	0.582	-
P (mg/L)	103.88	26.65	96.01	23.91	115.65	43.78	2.780	0.065	-
Na (mg/L)	2139.34	567.21	2035.64	548.37	2359.98	768.47	2.562	0.080	-
K (mg/L)	482.60	357.43	251.26	253.45	379.36	285.58	3.811	0.024	0.044 (0.000; 0.111)
Fe (mg/L)	1.57	0.56	1.59	0.77	1.60	0.65	0.034	0.967	-
Mg (mg/L)	17.54	7.14	17.20	5.03	19.11	6.19	1.341	0.264	-
Cu (mg/L)	1.09	0.42	0.89	0.32	1.13	0.44	2.247	0.109	-
Zn (mg/L)	2.78	2.04	2.76	1.77	2.44	1.05	1.085	0.340	-
Sr (mg/L)	0.14	0.06	0.17	0.08	0.14	0.07	1.325	0.269	-

M—mean, SD—standard deviation.

**Table 7:** The levels of bioelements in women with excess body weight and with normal body weight.

Bioelement Levels	Body Weight						F (2, 167)	*p* *	η2 (95%CI)
	Normal Weight (n = 55)		Overweight (n = 70)		Obesity (n = 44)				
	M	SD	M	SD	M	SD			
Ca (mg/L)	107.80	27.84	98.20	25.12	105.36	34.05	1.923	0.149	-
P (mg/L)	115.69	34.96	107.14	35.65	109.76	47.70	0.772	0.464	-
Na (mg/L)	2438.55	675.22	2102.83	576.82	2317.88	878.29	3.746	0.026	0.043 (0.000; 0.110)
K (mg/L)	422.61	307.11	331.93	269.32	456.71	354.24	2.597	0.078	-
Fe (mg/L)	1.72	0.65	1.49	0.58	1.59	0.67	1.995	0.139	-
Mg (mg/L)	20.20	6.77	17.17	4.90	18.45	7.44	3.638	0.028	0.042 (0.000; 0.108)
Cu (mg/L)	1.14	0.39	1.00	0.39	1.19	0.50	3.425	0.035	0.039 (0.000; 0.104)
Zn (mg/L)	2.56	1.07	2.51	1.56	2.64	1.69	0.106	0.899	-
Sr (mg/L)	0.14	0.07	0.15	0.07	0.14	0.06	0.481	0.619	-

* one-way ANOVA, M—mean, SD—standard deviation.

**Table 8:** The levels of bioelements in women with and without hypertension (HA).

Bioelement	HA (n = 39)		Non-HA (n = 131)		t_df = 168_	*p* *	Δ	95%CI
	M	SD	M	SD				
Ca (mg/L)	99.20	26.18	104.49	29.40	−1.011	0.314	5.29	−5.04; 15.63
P (mg/L)	102.25	21.73	113.19	42.38	−1.550	0.123	10.94	−2.99; 24.87
Na (mg/L)	2130.29	551.72	2312.96	746.30	−1.416	0.159	182.67	−71.93; 437.27
K (mg/L)	437.86	344.50	381.76	297.31	0.997	0.320	−56.10	−167.24; 55.04
Fe (mg/L)	1.60	0.63	1.59	0.64	0.090	0.928	−0.01	−0.24; 0.22
Mg (mg/L)	17.23	5.38	18.90	6.61	−1.440	0.152	1.67	−0.62; 3.96
Cu (mg/L)	1.01	0.31	1.12	0.45	−1.466	0.145	0.11	−0.04; 0.27
Zn (mg/L)	2.72	1.90	2.51	1.29	0.808	0.420	−0.21	−0.73; 0.31
Sr (mg/L)	0.15	0.07	0.14	0.06	1.033	0.303	−0.01	−0.04; 0.01

M—mean, SD—standard deviation.

**Table 9:** Correlations between the levels of bioelements in women.

Variables	Bioelements																	
	Ca (mg/L)		P (mg/L)		Na (mg/L)		K (mg/L)		Fe (mg/L)		Mg (mg/L)		Cu (mg/L)		Zn (mg/L)		Sr (mg/L)	
	r	*p*	r	*p*	r	*p*	r	*p*	r	*p*	r	*p*	r	*p*	r	*p*	r	*p*
FPG (mg/dL)	0.10	0.197	−0.07	0.387	−0.01	0.916	−0.09	0.227	−0.01	0.934	−0.01	0.916	−0.04	0.561	0.03	0.656	0.16	0.041
Insulin (µlu/mL)	−0.09	0.261	−0.13	0.080	−0.10	0.175	0.13	0.092	−0.09	0.227	−0.05	0.527	0.12	0.125	−0.03	0.733	−0.12	0.135
HbA1C (%)	0.09	0.244	−0.05	0.480	0.02	0.810	−0.14	0.079	−0.04	0.630	0.05	0.493	−0.11	0.147	−0.02	0.772	0.18	0.017
TC (mg/dL)	−0.05	0.511	−0.09	0.224	−0.12	0.133	0.03	0.737	0.00	0.997	−0.08	0.272	−0.18	0.022	0.06	0.421	0.10	0.207
TG (mg/dL)	0.06	0.457	−0.12	0.114	−0.07	0.354	−0.01	0.874	−0.02	0.837	0.08	0.318	−0.09	0.261	0.10	0.173	0.13	0.096
HDL (mg/dL)	0.09	0.247	0.02	0.801	0.10	0.198	0.02	0.788	0.11	0.165	−0.02	0.821	0.02	0.807	0.07	0.396	0.05	0.554
LDL (mg/dL)	−0.10	0.173	−0.08	0.288	−0.15	0.045	0.02	0.843	−0.05	0.540	−0.09	0.230	−0.16	0.042	0.00	0.978	0.06	0.451
WC (cm)	0.00	0.967	0.07	0.384	−0.02	0.828	0.08	0.323	0.04	0.632	−0.16	0.035	0.06	0.414	0.10	0.184	0.10	0.191
BMI	−0.04	0.626	−0.04	0.594	−0.07	0.335	−0.02	0.796	−0.09	0.257	−0.13	0.092	0.04	0.562	0.05	0.518	0.06	0.447
WHtr	0.02	0.763	0.10	0.186	0.01	0.896	0.09	0.259	0.05	0.480	−0.14	0.075	0.09	0.222	0.11	0.172	0.09	0.218
DBP (mmHg)	−0.08	0.316	−0.03	0.679	−0.09	0.261	0.02	0.795	−0.06	0.448	−0.13	0.086	−0.01	0.902	0.04	0.567	−0.01	0.914
SBP (mmHg)	−0.09	0.266	−0.14	0.064	−0.19	0.011	0.01	0.940	−0.08	0.313	−0.23	0.003	−0.17	0.029	0.13	0.087	0.12	0.133

*p*—statistical significance.

## Data Availability

Not applicable.

## Supplementary Materials

The following supporting information can be downloaded at: https://www.mdpi.com/article/10.3390/nu14194102/s1, Table S1. Analysis of reference material Seronorm™ Trace Elements Serum L-1, cat. 201405 using ICP-OES. Table S2. Reference range of bioelements in blood. Table S3. Post hoc comparison (WC, WHtR, SBP, DBP) with the control group. Table S4. Post hoc comparison (FPG, TG, HDL, TH/HDL, TC/HDL, LDL/HDL) with the control group. Table S5. Post hoc comparison (Na, Mg, Cu) with the control group.
