# Supplementary material for: The Levels of Bioelements in Postmenopausal Women with Metabolic Syndrome
Source: Nutrients. 2022 Oct 2;14(19):4102. doi: 10.3390/nu14194102 (PMC9572475; doi:10.3390/nu14194102)
Supplement: Supplementary file 1 [file nutrients-14-04102-s001.zip › nutrients-1907206-supplementary.pdf]

Table S1. Analysis of reference material Seronorm™ Trace Elements Serum L^{-1}, cat. 201405 using ICP-OES

Element	Seronorm™ Trace Elements Serum L^{-1} Certified [mg/L]	Seronorm™ Trace Elements Serum L^{-1} Measured [mg/L]	Recovery (%)
P 178.284	65.00	69.41	106.78
K 766.490	126.00	130.09	103.25
Ca 315.887	96.00	94.98	98.94
Fe 239.562	1.43	1.38	96.50
Na 589.592	2998.00	3105.12	103.57
Mg 285.213	20.60	22.49	109.17
Sr 421.552	0.026	0.031	119.23
Cu 224.700	1.88	1.67	89.89
Zn 206.200	1.74	1.91	109.77

Table S2. Reference range of bioelements in blood

Element	Reference range		
	mmol/L	mg/dL or mcg/dL	mg/L
Ca	2.15–2.55 mmol/L	9–10.5 mg/dL	90–105 mg/L
P	0.96–1.44 mmol/L	3–4.5 mg/dL	30–45 mg/L
Na	135–145 mmol/L	-	-
K	3.5–5.0 mmol/L	-	-
Fe	-	60–140 mcg/dL	0.6–1.4 mg/L
Mg	0.82–1.24 mmol/L	2–3 mg/dL	20–30 mg/L
Cu	-	85- 180 mcg/dL	0.85–1.8 mg/L
Zn	–	-	0.07–0.12 mg/L
Sr	-	0.0005–0.002 mg/dL	0.005–0.02 mg/L

Table S3. Post hoc comparison (WC, WHtR, SBP, DBP) with the control group

No.	Mets	Variables*			
		WC (cm)	WHtR	SBP (mmHg)	DBP (mmHg)
		1<br>86.734	1<br>52.816	1<br>111.30	1<br>74.220
1	no				
2	pre-Mets	0.0001	0.00006	0.00002	0.00002
3	Mets	0.05	0.07	0.0009	0.002

*Dunnett's test, probabilities for post-hoc tests (M > Control)

Table S4. Post hoc comparison (FPG, TG, HDL, TH/HDL, TC/HDL, LDL/ HDL) with the control group

No.	Mets	Variables*					
		FPG (mg/dL)	TG (mg/dL)	HDL (mg/dL)	TG/HDL ratio	TC/HDL ratio	LDL/HDL ratio
		1	1	1	1	1	1
		83.285	85.563	73.873	1.3232	2.9513	1.7027
1	no						
2	pre-Mets	0.044	0.002	0.025	0.02	0.0001	0.001
3	Mets	0.003	0.00002	0.00002	0.00002	0.00002	0.00002

*Dunnett's test, probabilities for post-hoc tests (M > Control)

Table S5 Post hoc comparison (Na, Mg, Cu) with the control group

No.	Mets	Variables*								
		Na (mg/L)			Mg (mg/L)			Cu (mg/L)		
		1	2	3	1	2	3	1	2	3
		2438.5	2102.8	2317.9	20.195	17.166	18.450	1.1408	0.99504	1.1898
1	normal weight		0.008	0.39		0.008	0.17		0.054	0.56
2	overweight	0.008		0.11	0.008		0.29	0.054		0.017
3	obesity	0.39	0.11		0.17	0.29		0.56	0.017	

**Fisher's* Least Significant Difference (LSD) test
